# Association between RBC folate and lumbar bone mineral density in postmenopausal women, a cross-sectional study from NHANES 2009–2018

**DOI:** 10.3389/fendo.2025.1559043

**Published:** 2025-04-28

**Authors:** Hua Wei, Ziqi Jin, Liangji Zhou, Gangjian Tang, Sheng Chai, Xiaoqiao Che, Yongxing Tan, Weiqing Zeng

**Affiliations:** ^1^ Department of Orthopedics, Guilin Municipal Hospital of Traditional Chinese Medicine, Guilin, Guangxi, China; ^2^ Graduate College, Guangxi University of Chinese Medicine, Nanning, Guangxi, China

**Keywords:** lumbar BMD, RBC folate, osteoporosis, postmenopausal female, a cross-sectional study, NHANES

## Abstract

**Background:**

Postmenopausal women are at an increased risk of bone density reduction, with multiple factors implicated, including folate, a B vitamin whose impact on bone health is gaining attention. The purpose of this research was to examine the association between red blood cell (RBC) folate levels and lumbar bone mineral density (BMD) in postmenopausal women.

**Methods:**

We performed a cross-sectional study to investigate the association between postmenopausal women’s lumbar BMD and RBC folate levels, using the data from the 2009–2018 National Health and Nutrition Examination Survey (NHANES). Participants were categorized into quartiles based on RBC folate levels (Q1-Q4). Univariate and multivariate regression models assessed the association between RBC folate levels and lumbar BMD, with threshold effect analysis performed.

**Results:**

A total of 1315 postmenopausal women were included. RBC folate levels were positively associated with lumbar BMD. The trend analysis across the quartiles of RBC folate indicated a statistically significant trend in all models (P for trend: Model 1 = 0.020; Model 2 = 0.015; Model 3 = 0.037), suggesting that higher RBC folate levels are associated with increased lumbar BMD. In the unadjusted model 1, a 10 nmol/L increase in RBC folate was associated with a 0.0002 g/cm² increase in lumbar BMD (P=0.002509). The correlation was still significant (P=0.0006) even after age and race were taken into account (model 2). Further adjustment for multiple variables (model 3) showed a 0.0002 g/cm² increase in lumbar BMD per 10 nmol/L increase in RBC folate (P=0.0212). Threshold effect analysis revealed a breakpoint at 92.4 nmol/dL, suggesting a nonlinear relationship between RBC folate levels and lumbar BMD.

**Conclusions:**

Postmenopausal women’s RBC folate levels had a positive association with their lumbar BMD. Maintaining appropriate RBC folate levels may help preserve bone density and offer a fresh approach to avoiding osteoporosis in postmenopausal women.

## 1 Introduction

The symptoms of osteoporosis, a systemic skeletal illnesses, involve decreased bone mass, altered bone microstructure, increased bone fragility, and a higher risk of fractures (1, 2). Predominantly affecting postmenopausal women, this condition often stems from estrogen deficiency, which is linked to decreased bone density (3, 4). The impact of osteoporosis on the quality of life is substantial, particularly among women who have undergone menopause (5, 6). It is the most prevalent disease in women and ranks second among men, with postmenopausal women having a higher incidence of hip fractures than men (7). Nutritional factors are recognized as pivotal in influencing bone density (8). Folate, or vitamin B9, is a crucial dietary component for skeletal health maintenance (9). Elevated homocysteine (Hcy) levels are identified as an independent risk factor for osteoporosis, with folate deficiency correlating to increased Hcy levels (10). Research indicates that micronutrient supplementation containing folate can modulate Hcy levels in older adult individuals (11–13). Folate supplementation is primarily achieved through balanced diets and supplements. While most research have focused on the association between folate intake and bone mineral density (BMD) or osteoporosis, fewer have studied the link between red blood cell folate and bone density (14–16). A study has demonstrated a positive correlation between serum folate levels and BMD (17). However, serum folate levels are less stable than red blood cell folate levels, necessitating further research into the relationship between red blood cell folate and BMD in postmenopausal women. Such studies could potentially enhance our understanding of bone density improvement and osteoporosis prevention, which may also contribute to the development of osteoporosis screening strategies for postmenopausal women.

## 2 Materials and methods

### 2.1 Study population

The National Health and Nutrition Examination Survey (NHANES) database, which encompasses comprehensive health and nutrition data on the US population, served as the source of data for this study’s analysis. The survey findings have been instrumental in shaping health programs and services, enhancing health awareness, and assessing the prevalence of significant diseases and illness risk factors. For our analysis, we integrated data from five consecutive two-year cycles of the NHANES, conducted from 2009 to 2018. All procedures were approved by the National Centre for Health Statistics Research Ethics Review Board, and participants presented their informed consent for the use of anonymized data in research.

**Figure 1:**
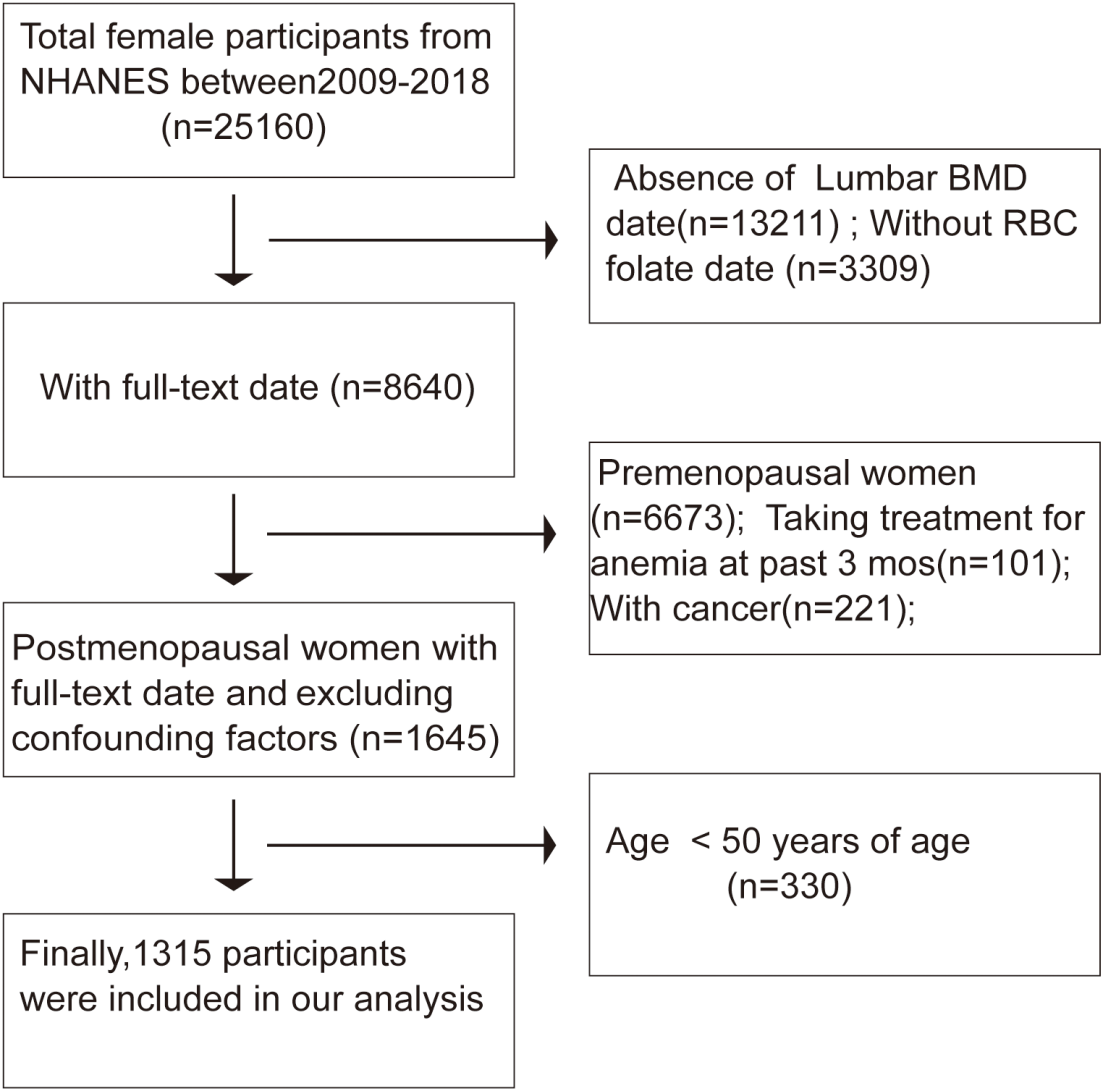
Flowchart of sample selection from the NHANES 2009-2018.

With the complete de-identified dataset, the current study was deemed exempt by the institutional review committee of the author’s institution. The study was carried out in accordance with the Helsinki Declaration. The participants in our research had to be at least 50 years old, specifically postmenopausal women, with the NHANES database yielding 1315 eligible patients during the 2009–2018 period. Out of 25,160 female participants in the survey, those lacking lumbar bone mineral density (BMD) data (n = 13,211) and those with uncertain RBC folate status (n = 3,309) were excluded. Menopausal status was ascertained based on responses to a reproductive health questionnaire. Participants were initially asked, “Have you had regular periods in the past 12 months?” Those who answered “no” were further inquired, “What is the reason for not having regular periods? (Options: Menopause/change of life; Pregnancy; Breastfeeding; Medical conditions/treatments; other)” Ultimately, 1,967 postmenopausal women were included in enrollment. Additionally, individuals who had received anemia treatment within the past three months (n = 101) or those with missing cancer values (n = 221) were not included. Consistent with previous studies (17), participants under 50 years old (n = 330) were also excluded. After screening, our analysis included data from 1,315 participants (**Figure 1**). A synopsis of the NHANES survey’s data-gathering techniques is available at www.cdc.gov/nchs/nhanes/. The Mobile Examination Center was used for physical and medical examinations, as well as the collecting of urine and blood samples. Data on lifestyle, health, and demographics was gathered through in-home interviews.

### 2.2 Folate status

Folate status in the population was assessed differently across the NHANES cycles from 2009 to 2018. For the 2009–2010 NHANES, a microbiologic assay was employed to measure whole-blood and serum folate levels, from which red blood cell folate (RBC folate) was estimated. In contrast, the folate status assessment for the 2011–2018 NHANES cycles integrated two analytical methods: serum folate levels were determined using isotope-dilution high-performance liquid chromatography- tandem mass spectrometry (LC-MS/MS), while whole-blood folate levels were ascertained through microbiologic testing. By synthesizing data from these two methodologies, RBC folate concentrations were subsequently calculated. {whole blood folate−[serum blood folate∗(1.0 − hematocrit/100)]}/(hematocrit/100) is the equation for red blood cell folate.

### 2.3 Covariates

The NHANES database provided additional information on potential confounders that were accounted for in our analysis. A Hologic QDR-4500A densitometer was used to measure lumbar bone mineral density (BMD), and qualified radiology technologists used established procedures. Continuous variables included age, Poverty Income Ratio (PIR), serum iron, calcium, vitamin D, total protein, total cholesterol, blood urea nitrogen, serum creatinine, and total bilirubin. Categorical variables were classified as follows: race, smoking status (defined as having smoked 100 cigarettes or more over one’s lifetime), intensity of work activity, educational level, alcohol intake (defined as having had at least four drinks in one’s life), marital status, and body mass index (BMI), which was categorized into three categories: “normal” (BMI < 25 kg/m^2^), “overweight” (25 ≤ BMI < 30 kg/m^2^), and “obese” (BMI ≥ 30 kg/m^2^). For continuous variables with missing values, we used mean substitution. For categorical variables, we created a separate category for those with many missing values and merged those with few missing values into an “other” category. Further details on the collection methods for RBC folate status, lumbar BMD, and other variables can be obtained on the NHANES website(http://www.cdc.gov/nchs/nhanes/).

### 2.4 Statistical analysis

Statistical analyses adhered to NHANES guidelines, accounting for the survey’s complex sampling design to mitigate biases from sample selection, oversampling, and nonresponse. Unweighted multiple regression analyses were conducted to describe the association between RBC folate and LBMD, with the aim of identifying the corresponding β coefficient and its 95% confidence interval (CI). To facilitate a more comprehensive analysis, RBC folate levels were processed in two distinct ways: by categorizing them into four quartiles (Q1-Q4) and by performing logarithmic transformation to treat them as a continuous variable. For continuous variables, presented as weighted means ± standard, weighted t-tests were utilized to examine differences in RBC folate levels among participants across the different quartiles. In the analysis of categorical variables, presented as weighted percentages, weighted chi-square tests were employed. For inter-group comparisons, the first quartile (Q1) was chosen as the baseline characteristics group. Additionally, to ensure the robustness of our findings, the second quartile (Q2) was considered as a back-up baseline feature. In constructing the regression models, adherence to the Strengthening the Reporting of Observational Studies in Epidemiology (STROBE) standards was strictly observed (18). Three regression models were designed as follows: Model 1 was the unadjusted base model; Model 2 incorporated adjustments for race and age; and Model 3 included comprehensive adjustments for all other covariates. To further explore potential moderating factors, subgroup analyses were conducted, stratifying the data by race, educational level, and smoking status. Additionally, we employed a piecewise linear regression model to determine possible inflection points. All statistical analyses were performed using EmpowerStats (version 5.0) and R (version 3.4.3), with a p-value of less than 0.05 considered statistically significant.

## 3 Results

### 3.1 Baseline characteristics

**Table 1** provides specifics on the baseline characteristics of the 1,315 research participants. Participants’ red blood cell folate levels were divided into four quartiles. Compared to the first quartile (Q1), individuals in the higher quartile groups exhibited significantly higher levels of serum vitamin D, blood urea nitrogen, and PIR. Additionally, a larger proportion of participants in these quartiles identified as Non-Hispanic White. Furthermore, a greater percentage of participants in the upper quartiles reported never having smoked 100 cigarettes or more over one’s lifetime.

**Table 1:** Basic characteristics of 1315 participants.

RBC folate(nmol/L) quartile	Q1	Q2	Q3	Q4	P-value
N	329	315	335	336	
Serum vitamin D (nmol/L)	56.23 ± 27.03	58.07 ± 23.34	69.64 ± 27.04	83.88 ± 30.60	<0.001
Age (years)	57.65 ± 6.55	57.56 ± 6.21	57.20 ± 6.20	59.58 ± 8.19	<0.001
The ratio of income to poverty	2.30 ± 1.51	2.57 ± 1.56	2.68 ± 1.55	2.80 ± 1.58	<0.001
BMI (kg/m2)(%)	30.17 ± 7.53	31.01 ± 7.36	29.19 ± 6.41	30.35 ± 6.82	0.011
Blood urea nitrogen (mmol/L)	4.76 ± 1.79	4.85 ± 1.65	4.85 ± 1.72	5.14 ± 1.80	0.034
Calcium (mmol/L)	2.36 ± 0.09	2.36 ± 0.09	2.37 ± 0.09	2.37 ± 0.09	0.209
Serum cholesterol (mmol/L)	5.51 ± 1.19	5.45 ± 1.10	5.39 ± 1.02	5.45 ± 1.06	0.548
Serum creatinine (umol/L)	68.14 ± 18.15	69.32 ± 32.21	68.79 ± 30.42	71.52 ± 17.39	0.340
Serum Iron (umol/L)	14.30 ± 5.32	13.79 ± 4.79	14.56 ± 5.38	13.90 ± 5.06	0.198
Total protein (g/L)	72.23 ± 4.79	72.05 ± 4.41	71.39 ± 5.05	70.92 ± 4.41	<0.001
Lumbar BMD (g/cm2)	0.93 ± 0.16	0.95 ± 0.16	0.95 ± 0.15	0.97 ± 0.15	0.032
Total bilirubin (umol/L)	9.95 ± 3.93	10.31 ± 4.60	10.48 ± 3.84	10.40 ± 3.93	0.360
Race (%)					<0.001
Non-Hispanic White	73 (22.19%)	89 (28.25%)	136 (40.60%)	187 (55.65%)	
Non-Hispanic Black	122 (37.08%)	86 (27.30%)	62 (18.51%)	39 (11.61%)	
Mexican American	66 (20.06%)	58 (18.41%)	51 (15.22%)	41 (12.20%)	
Other race	68 (20.67%)	82 (26.03%)	86 (25.67%)	69 (20.54%)	
Educational level (%)					0.256
Less than some college	174 (52.89%)	168 (53.33%)	163 (48.66%)	154 (45.83%)	
Some college	100 (30.40%)	83 (26.35%)	98 (29.25%)	111 (33.04%)	
More than college	55 (16.72%)	64 (20.32%)	74 (22.09%)	71 (21.13%)	
Living with parter					0.039
Yes	167 (50.76%)	167 (53.02%)	199 (59.40%)	201 (59.82%)	
No	162 (49.24%)	148 (46.98%)	136 (40.60%)	135 (40.18%)	
Having at least 4 drinks in life (%)					0.926
Yes	43 (13.07%)	37 (11.75%)	37 (11.04%)	37 (11.01%)	
No	146 (44.38%)	146 (46.35%)	157 (46.87%)	165 (49.11%)	
Missing	140 (42.55%)	132 (41.90%)	141 (42.09%)	134 (39.88%)	
Vigorous work activity (%)					0.510
Yes	44 (13.37%)	32 (10.16%)	37 (11.04%)	34 (10.12%)	
No	285 (86.63%)	283 (89.84%)	298 (88.96%)	302 (89.88%)	
Smoking status (%)					0.268
Yes	145 (44.07%)	124 (39.37%)	129 (38.51%)	124 (36.90%)	
No	184 (55.93%)	191 (60.63%)	206 (61.49%)	212 (63.10%)	

Mean ± SD for continuous variables: P value was calculated by the weighted linear regression model.% for categorical variables: P value was calculated by the weighted chi-square test.

### 3.2 Associations of RBC folate status with LBMD

As presented in **Table 2**, the trend analysis across quartiles of RBC folate levels revealed a statistically significant increasing trend in all models for the association with lumbar bone mineral density (BMD) (P for trend: Model 1 = 0.020; Model 2 = 0.015; Model 3 = 0.037). This suggests that higher RBC folate levels are linked to greater lumbar BMD. However, when Q2 was used as the reference category instead of Q1, the association’s significance was altered.

**Table 2:** Associations of RBC folate with lumbar bone mineral density.

	Model 1β (95%CI, P)	Model 2β (95%CI, P)	Model 3β (95%CI, P)
RBC folate quartiles (nmol/L)			
Q1 (114-833)	Reference	Reference	Reference
Q2 (834-1090)	0.0382 (0.0042, 0.0721) 0.0305	0.0405 (0.0071, 0.0739) 0.0202	0.0280 (-0.0004, 0.0564) 0.0609
Q3 (1100-1460)	0.0239 (-0.0056, 0.0535) 0.1161	0.0279 (-0.0008, 0.0567) 0.0607	0.0266 (0.0003, 0.0529) 0.0543
Q4 (1470-4580)	0.0347 (0.0048, 0.0647) 0.0260	0.0413 (0.0107, 0.0720) 0.0101	0.0294 (0.0001, 0.0588) 0.0565
P for trend	0.020	0.015	0.037

Model 1: no covariates were adjusted.

Model 2: age, race were adjusted.

Model 3: age, race, the ratio of income to poverty, serum iron, calcium, vitamin D, total protein, total cholesterol, blood urea nitrogen, serum creatinine, living with parter, total bilirubin, smoking at least 100 cigarettes in life, vigorous work activity, educational level, drinking at least 3 alcohol over past 12 mos and BMI were adjusted.

Subgroup analyses by race, educational level, and smoking status were conducted, and the results were consistent, as shown in **Table 3**. In the unadjusted model, lumbar BMD increased by 0.0002 g/cm² for every 10 nmol/L rise in RBC folate (P = 0.0025). When age and race were taken into account, this relationship was still significant (Adjust I model, P = 0.0006). Further adjustment for additional variables (Adjust II model) retained the significance, with a 0.0002 g/cm² increase in lumbar BMD for each 10 nmol/L increase in RBC folate (P = 0.0212), although the statistical significance was somewhat reduced.

**Table 3:** Association of RBC folate with lumbar BMD,stratified by race, educational level or smoking.

	Model 1β (95%CI, P)	Model 2 β (95%CI, P)	Model 3 β (95%CI, P)
RBC folate (nmol/dL)	0.0002 (0.0001, 0.0004) 0.0025	0.0002 (0.0001, 0.0004) 0.0006	0.0002 (0.0000, 0.0003) 0.0212
Stratified by Race			
Non-Hispanic White	0.0001 (-0.0001, 0.0003) 0.2459	0.0002 (-0.0001, 0.0004) 0.1474	0.0001 (-0.0002, 0.0003) 0.5873
Non-Hispanic Black	0.0005 (0.0001, 0.0009) 0.0220	0.0005 (0.0001, 0.0009) 0.0165	0.0004 (-0.0000, 0.0008) 0.0714
Mexican American	0.0006 (0.0002, 0.0011) 0.0052	0.0006 (0.0002, 0.0011) 0.0054	0.0008 (0.0003, 0.0013) 0.0033
Other race	0.0007 (0.0004, 0.0010) < 0.0001	0.0007 (0.0004, 0.0010) < 0.0001	0.0006 (0.0003, 0.0010) 0.0006
Stratified by Educational level			
Less than some college	0.0002 (-0.0000, 0.0004) 0.0550	0.0002 (0.0000, 0.0004) 0.0356	0.0001 (-0.0001, 0.0003) 0.2850
Some college	0.0002 (-0.0001, 0.0004) 0.1551	0.0002 (-0.0000, 0.0004) 0.1012	0.0002 (-0.0001, 0.0004) 0.2124
More than college	0.0003 (0.0000, 0.0007) 0.0482	0.0004 (0.0001, 0.0007) 0.0231	0.0002 (-0.0001, 0.0006) 0.2329
Stratified by Smoking status			
Yes	0.0004 (0.0001, 0.0006) 0.0020	0.0004 (0.0002, 0.0007) 0.0004	0.0002 (-0.0000, 0.0005) 0.0939
No	0.0001 (-0.0001, 0.0003) 0.2097	0.0001 (-0.0001, 0.0003) 0.1713	0.0001 (-0.0000, 0.0003) 0.1071

Model 1: no covariates were adjusted.

Model 2: age, race were adjusted.

Model 3: age, race, the ratio of income to poverty, serum iron, calcium, vitamin D, living with parter, total protein, total cholesterol, blood urea nitrogen, serum creatinine, total bilirubin, smoking at least 100 cigarettes in life, vigorous work activity, educational level, drinking at least 3 alcohol over past 12 mos and BMI were adjusted.In the subgroup analysis, the model is not adjusted for the stratification variable itself.

**Figure 2:**
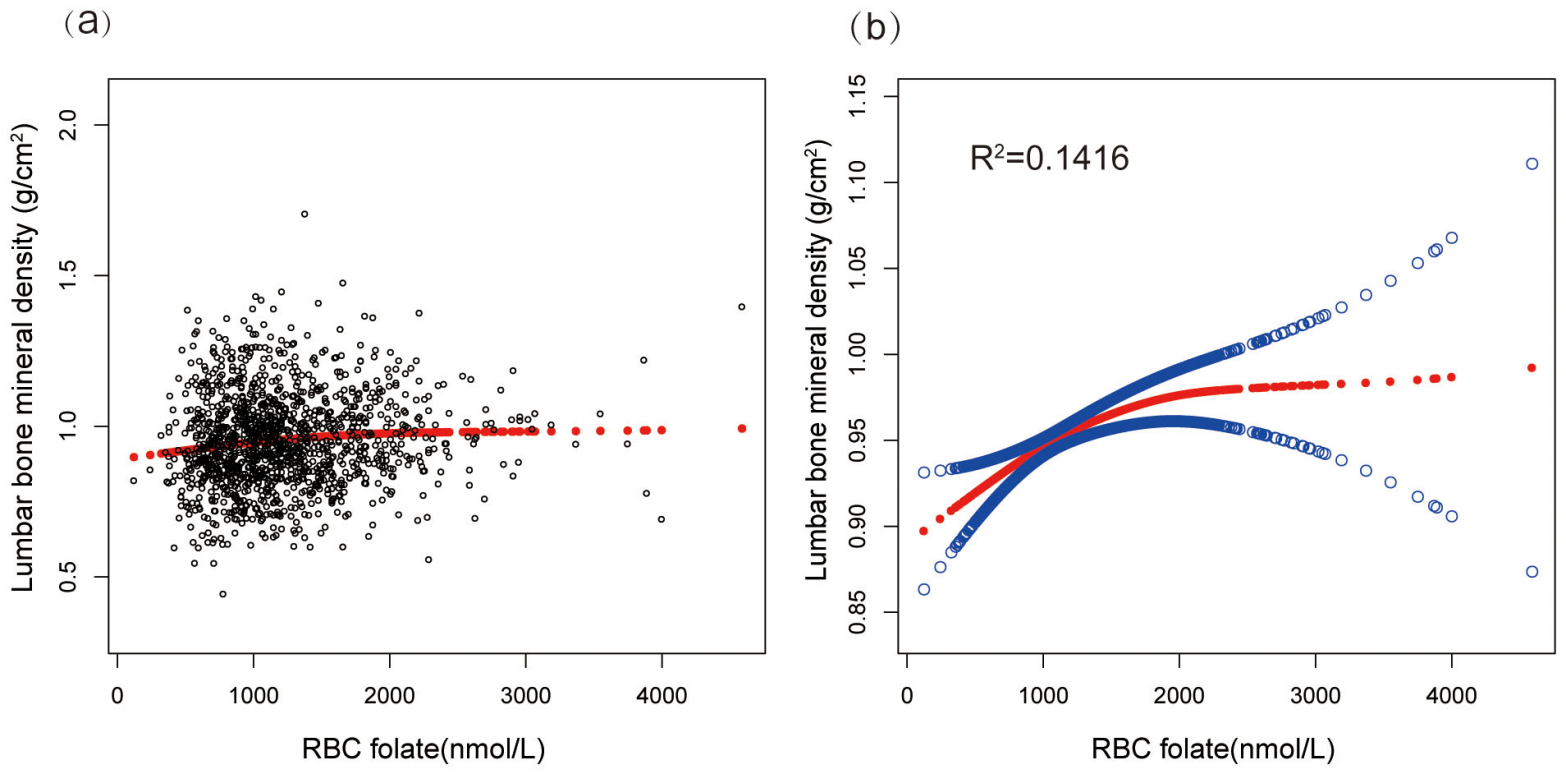
A scatter plot and smooth curve fitting illustrated the association between serum RBC folate level and lumbar bone mineral density. **(a)** Each black point represents a sample. **(b)** Solid red line represents the smooth curve fit between variables (R^2^=0.1416). Blue bands represents the 95% of confidence interval from the fit. Adjusted for age, race, the ratio of income to poverty, serum iron, calcium, vitamin D, total protein, total cholesterol, blood urea nitrogen, serum creatinine, living with parter, total bilirubin, smoking at least 100 cigarettes in life, vigorous work activity, educational level, drinking at least 3 alcohol over past 12 mos and BMI.

Furthermore, the lumbar BMD of postmenopausal women showed a U-shaped association with erythrocyte folate levels (**Figure 2**). To explore the potential non-linear relationship between erythrocyte folate levels and lumbar BMD, we utilized smooth curve fitting techniques. The analysis identified a breakpoint at 92.4 nmol/dL, with effects of 0.0011 (P = 0.0015) below this threshold and 0.0002 (P = 0.1083) above it. These findings suggest a potential non-linear effect of RBC folate levels on lumbar BMD, as detailed in **Table 4**.

**Table 4:** Threshold effect analysis between RBC folate and lumbar BMD in postmenopausal women using a two-piecewise linear regression model.

	Adjusted β (95% CI), p-value
RBC folate (nmol/dL)	
Aged 40-50 years	
Fitting by standard linear model	0.0003 (0.0001, 0.0005) 0.0005
Fitting by two-piecewise linear model	
(K)Inflection point	92.4
RBC folate < 92.4(nmol/dL)	0.0011 (0.0004, 0.0018) 0.0015
RBC folate > 92.4 (nmol/dL)	0.0002 (-0.0000, 0.0004) 0.1083
Log-likelihood ratio	0.015

Age, race, living with parter, the ratio of income to poverty, serum iron, calcium, vitamin D, total protein, total cholesterol, blood urea nitrogen, serum creatinine, total bilirubin, smoking at least 100 cigarettes in life, vigorous work activity, educational level, living with parter, drinking at least 3 alcohol over past 12 mos and BMI were adjusted.

## 4 Discussion

This study provides clear evidence that lumbar BMD in postmenopausal women is positively correlated with RBC folate levels. Our findings indicate that elevated RBC folate levels are linked to higher lumbar BMD, a relationship that remains significant even after accounting for various confounding factors. Although the significance of some associations was attenuated after multivariable adjustments, the overall trend suggests a potential role for folate in influencing bone mineral density. Additionally, a threshold effect study showed a nonlinear connection with an inflection point at 92.4 nmol/dL, suggesting that the impact of RBC folate on BMD may vary depending on the content of folate. These results underscore the importance of adequate folate levels for bone health and hint at the possibility that interventions to increase folate intake could be particularly beneficial for postmenopausal women with lower RBC folate levels.

Osteoporosis is a significant global health concern, especially among postmenopausal women, due to the heightened risk of fractures and the associated morbidity and mortality (19). Identifying modifiable dietary factors like folate that impact bone health is crucial for developing effective prevention strategies. Folate, an essential B vitamin, is involved in numerous physiological processes, including DNA synthesis, methylation, and homocysteine (Hcy) metabolism, all of which may influence bone health (20). Red blood cell folate content, which reaches a median equilibrium of 9 months following the initiation of folic acid supplementation, allows for a more comprehensive assessment of folate consumption over time (21). RBC folate are regarded to be a trustworthy indication of tissue folate levels (22, 23). This study found a significant positive association between RBC folate levels and lumbar BMD in postmenopausal women, particularly at lower folate levels. The identified inflection point at 92.4 nmol/dL suggests that while folate is beneficial for bone health, its protective effects may plateau or diminish at higher concentrations. These findings stress the importance of maintaining sufficient folate levels for bone health in postmenopausal women and suggest that interventions aimed at increasing folate intake could be especially beneficial for those with lower RBC folate levels.

Folate’s role in bone health has been increasingly recognized, particularly in postmenopausal women who are more susceptible to osteoporosis. Studies suggest that folate may protect bones through several molecular mechanisms, including Hcy regulation and oxidative stress reduction (24, 25). Hcy, a sulfur-containing amino acid and an intermediate in methionine metabolism, has been implicated in bone loss and increased fracture risk when present at elevated levels, a condition known as hyperhomocysteinemia (26). When methionine synthase catalyzes the remethylation of Hcy to methionine with vitamin B12 as a cofactor, folate is an essential component (24, 25). This process lowers circulating Hcy levels, thereby reducing its detrimental effects on bone. Elevated Hcy levels have been shown to induce oxidative stress and apoptosis in osteoblasts, the cells responsible for bone formation, while promoting osteoclastogenesis, leading to increased bone resorption (27, 28). Additionally, by decreasing nitric oxide bioavailability and elevating oxidative stress, Hcy causes endothelial dysfunction, which hinders blood flow to bone tissue and may jeopardize bone remodeling and repair (29).

Adequate folate levels are essential for maintaining low Hcy concentrations. Studies have shown that folate supplementation can significantly reduce plasma Hcy levels, thereby protecting against bone loss (30). A study by He et al. (2021) demonstrated that folic acid supplementation in a high-fat diet-induced osteoporosis mouse model reduced Hcy levels and improved bone microarchitecture through the activation of the AMPK signaling pathway (27). Clinical trials have also reported that folate supplementation, in combination with vitamin B12, effectively lowers Hcy levels and improves BMD in postmenopausal women (31). Another mechanism may involve reducing oxidative stress. Particularly in postmenopausal women, oxidative stress which is defined as an imbalance between the generation of reactive oxygen species (ROS) and antioxidant defenses is a significant cause of bone loss (25). ROS can directly damage bone cells and the extracellular matrix, as well as disrupt signaling pathways involved in bone remodeling. The antioxidant qualities of folate may be essential for reducing oxidative stress and maintaining bone health. The balance between osteoblasts (bone creation) and osteoclasts (bone resorption) is upset by excessive ROS generation because it causes osteoblast death and activates the RANKL signaling pathway, which encourages osteoclast development and activity and increases bone resorption (32, 33). ROS also degrades type I collagen and other components of the bone matrix, compromising bone strength and integrity (34). Folate contributes to oxidative stress reduction through glutathione synthesis, regulation of antioxidant enzymes, and mitochondrial protection (30). Folate is involved in the synthesis of glutathione, a major intracellular antioxidant that neutralizes ROS and protects bone cells from oxidative damage (35). Supplementing with folate has been demonstrated to increase the expression of antioxidant enzymes that scavenge reactive oxygen species (ROS) and lessen oxidative stress, including catalase and superoxide dismutase (SOD) (36). By regulating ROS generation and preserving mitochondrial membrane potential, folate maintains mitochondrial function and inhibits osteoblast apoptosis (37). Folate treatment decreased oxidative stress indicators and increased bone mineral density (BMD) in a model of postmenopausal osteoporosis, according to a research by Asbaghi et al. (38). The authors attributed these effects to enhanced glutathione synthesis and reduced RANKL expression (39). Clinical data from postmewer levels of oxidative stress biomarkers and improved bone health outcomes (40). The interplanopausal women have also shown that higher dietary folate intake is associated with loy between Hcy regulation and oxidative stress reduction highlights the multifaceted role of folate in bone health. Despite these possibilities, further research is needed to elucidate the underlying molecular mechanisms for the association between RBC folate and BMD and to develop effective interventions to prevent and treat osteoporosis.

This study has several strengths, including its nationally representative sample, the relatively stable folate status assessed, and the investigation of non-linear correlations using smooth curve fits. Nonetheless, it is critical to acknowledge some limitations. First, we cannot completely rule out the possibility of residual confounding that may affect RBC folate levels, even though we accounted for the majority of known confounders. Second, the absence of weighted regression analysis may mean that our results do not accurately reflect the relationship between red blood cell folate and lumbar BMD. Third, in line with other published research (41), we excluded patients whose age at the onset of osteoporosis was less than 50 years old, which may have introduced some statistical bias. Fourth, we only selected lumbar spine bone density for data comparison, which may not be comprehensive (42). However, many studies have made comparisons in this manner (43, 44). Fifth, causality between RBC folate levels and BMD cannot be established due to the cross-sectional character of the study, and longitudinal investigations are required to demonstrate the directionality of this connection (45, 46). Folate status, influenced by dietary intake, supplementation, and genetic factors, can vary significantly over an individual’s lifetime (47). Lastly, although the study controlled for multiple confounders, other factors such as genetic predispositions, specific dietary patterns, and gut microbiota composition may also influence the relationship between folate and BMD (48).

## 5 Conclusion

In conclusion, postmenopausal women’s RBC folate levels had a positive association with their lumbar BMD. Maintaining appropriate RBC folate levels may help preserve bone density and offer a fresh approach to avoiding osteoporosis in postmenopausal women.

## Data Availability

The datasets presented in this study can be found in online repositories. The names of the repository/repositories and accession number(s) can be found in the article/**Supplementary Material**.

## Supplementary material

Supplementary Table 1 Association of RBC folate with lumbar BMD, stratified by gender.

Supplementary Table 2 Association of RBC folate with lumbar BMD,stratified by age.

Supplementary Table 3 Association of RBC folate with lumbar BMD adjusting serum vitamin D.

Supplementary Table 4 Association of RBC folate with serum vitamin D.

Supplementary Table 5 Association of RBC folate with lumbar BMD,stratified by Vigorous work activity.

Supplementary Table 6 Association of RBC folate with lumbar BMD in different segments.
