# Supplementary material for: Association between RBC folate and lumbar bone mineral density in postmenopausal women, a cross-sectional study from NHANES 2009–2018
Source: Front Endocrinol (Lausanne). 2025 Apr 28;16:1559043. doi: 10.3389/fendo.2025.1559043 (PMC12066272; doi:10.3389/fendo.2025.1559043)
Supplement: Supplementary Table 1 — Association of RBC folate with lumbar BMD, stratified by gender. [file Table1.doc]

**Supplementary Table 1 Association of RBC folate with lumbar BMD, stratified by gender**

| Gender | Model1β (95%CI, P) | Model2 β (95%CI, P) | Model 3 β (95%CI, P) |
| --- | --- | --- | --- |
| Male | 0.0002 (-0.0000, 0.0003) 0.0547 | 0.0002 (0.0000, 0.0004) 0.0169 | 0.0001 (-0.0001, 0.0002) 0.5285 |
| Female | 0.0003 (0.0001, 0.0004) 0.0016 | 0.0004 (0.0002, 0.0005) <0.0001 | 0.0003 (0.0001, 0.0005) 0.0002 |

Model 1: no covariates were adjusted.

Model 2: age, race were adjusted.

Model 3: age, race, living with parter, the ratio of income to poverty, serum iron, calcium, vitamin D, total protein, total cholesterol, blood urea nitrogen, serum creatinine, total bilirubin, smoking at least 100 cigarettes in life, vigorous work activity, educational level, living with parter, drinking at least 3 alcohol over past 12 mos and BMI were adjusted.

**Supplementary Table 2 Association of RBC folate with lumbar BMD ,stratified by age**

| Age | Model1β (95%CI, P) | Model2 β (95%CI, P) | Model 3 β (95%CI, P) |
| --- | --- | --- | --- |
| Age= <60 | 0.0003 (0.0001, 0.0005) 0.0018 | 0.0003 (0.0001, 0.0005) 0.0006 | 0.0003 (0.0001, 0.0005) 0.0006 |
| Age >=60, <70 | 0.0004 (0.0001, 0.0008) 0.0249 | 0.0003 (-0.0000, 0.0007) 0.0762 | 0.0002 (-0.0002, 0.0006) 0.3016 |
| Age >=70 | 0.0002 (-0.0003, 0.0007) 0.5169 | 0.0004 (-0.0002, 0.0009) 0.1855 | 0.0002 (-0.0004, 0.0008) 0.5276 |

Model 1: no covariates were adjusted.

Model 2: age, BMI were adjusted.

Model 3: race, living with parter, the ratio of income to poverty, serum iron, calcium, vitamin D, total protein, total cholesterol, blood urea nitrogen, serum creatinine, total bilirubin, smoking at least 100 cigarettes in life, vigorous work activity, educational level, living with parter, drinking at least 3 alcohol over past 12 mos and BMI were adjusted.

**Supplementary Table3 Association of RBC folate with lumbar BMD adjusting serum vitamin D**

| Gender | Model1β (95%CI, P) | Model2 β (95%CI, P) | Model 3 β (95%CI, P) | Model 4 β (95%CI, P) |
| --- | --- | --- | --- | --- |
| Male | 0.0002 (-0.0000, 0.0003) 0.0547 | 0.0002 (-0.0000, 0.0003) 0.0681 | 0.0001 (-0.0001, 0.0003) 0.2442 | 0.0001 (-0.0001, 0.0002) 0.5119 |
| Female | 0.0003 (0.0001, 0.0004) 0.0016 | 0.0003 (0.0002, 0.0005) 0.0002 | 0.0003 (0.0001, 0.0004) 0.0004 | 0.0003 (0.0001, 0.0005) 0.0002 |

Model 1: no covariates were adjusted.

Model 2: serum vitamin D was adjusted.
Model 3: age, race, living with parter, the ratio of income to poverty, serum iron, calcium, total protein, total cholesterol, blood urea nitrogen, serum creatinine, total bilirubin, smoking at least 100 cigarettes in life, vigorous work activity, educational level, living with parter, drinking at least 3 alcohol over past 12 mos and BMI were adjusted.
Model 4: age, race, living with parter, the ratio of income to poverty, serum iron, calcium, serum vitamin D, total protein, total cholesterol, blood urea nitrogen, serum creatinine, total bilirubin, smoking at least 100 cigarettes in life, vigorous work activity, educational level, living with parter, drinking at least 3 alcohol over past 12 mos and BMI were adjusted.

**Supplementary Table4 Association of RBC folate with serum vitamin D**

| Gender | Model1β (95%CI, P) | Model2 β (95%CI, P) | Model 3 β (95%CI, P) |
| --- | --- | --- | --- |
| Male | 0.1569 (0.1335, 0.1803) <0.0001 | 0.1239 (0.1006, 0.1473) <0.0001 | 0.1087 (0.0853, 0.1321) <0.0001 |
| Female | 0.2061 (0.1785, 0.2336) <0.0001 | 0.1707 (0.1424, 0.1990) <0.0001 | 0.1717 (0.1438, 0.1996) <0.0001 |

Model 1: no covariates were adjusted.

Model 2: age, race were adjusted.

Model3: Age, race, living with parter, the ratio of income to poverty, serum iron, calcium, serum vitamin D, total protein, total cholesterol, blood urea nitrogen, serum creatinine, total bilirubin, smoking at least 100 cigarettes in life, vigorous work activity, educational level, living with parter, drinking at least 3 alcohol over past 12 mos and BMI were adjusted.

**Supplementary Table 5 Association of RBC folate with lumbar BMD ,stratified by Vigorous work activity**

| Vigorous work activity | Model1β (95%CI, P) | Model2 β (95%CI, P) | Model 3 β (95%CI, P) |
| --- | --- | --- | --- |
| Yes | 0.0002 (-0.0003, 0.0006) 0.4706 | 0.0004 (-0.0000, 0.0009) 0.0810 | 0.0002 (-0.0003, 0.0008) 0.3994 |
| No | 0.0003 (0.0001, 0.0004) 0.0021 | 0.0003 (0.0002, 0.0005) <0.0001 | 0.0003 (0.0001, 0.0005) 0.0005 |

Model 1: no covariates were adjusted.

Model 2: age, race were adjusted.

Model 3: Age, race, living with parter, the ratio of income to poverty, serum iron, calcium, serum vitamin D, total protein, total cholesterol, blood urea nitrogen, serum creatinine, total bilirubin, smoking at least 100 cigarettes in life, educational level, living with parter, drinking at least 3 alcohol over past 12 mos and BMI were adjusted.

**Supplementary Table 6 Association of RBC folate with lumbar BMD in different segments**

| different segments | Model1β (95%CI, P) | Model2 β (95%CI, P) | Model 3 β (95%CI, P) |
| --- | --- | --- | --- |
| L1BMD | 0.0001 (-0.0000, 0.0002) 0.1520 | 0.0002 (0.0000, 0.0003) 0.0106 | 0.0001 (0.0000, 0.0003) 0.0349 |
| L2BMD | 0.0001 (0.0000, 0.0003) 0.0353 | 0.0002 (0.0001, 0.0003) 0.0030 | 0.0002 (0.0000, 0.0003) 0.0084 |
| L3BMD | 0.0001 (0.0000, 0.0003) 0.0282 | 0.0002 (0.0001, 0.0003) 0.0039 | 0.0002 (0.0000, 0.0003) 0.0163 |
| L4BMD | 0.0001 (-0.0000, 0.0002) 0.1868 | 0.0001 (0.0000, 0.0003) 0.0449 | 0.0001 (-0.0000, 0.0003) 0.0645 |

Model 1: no covariates were adjusted.

Model 2: age, race were adjusted.

Model 3: Age, race, living with parter, the ratio of income to poverty, serum iron, calcium, serum vitamin D, total protein, total cholesterol, blood urea nitrogen, serum creatinine, total bilirubin, smoking at least 100 cigarettes in life, vigorous work activity, educational level, living with parter, drinking at least 3 alcohol over past 12 mos and BMI were adjusted.
